# Factors influencing waist circumference among urban bank employees in Northeast Ethiopia: a cross-sectional study

**DOI:** 10.3389/fnut.2024.1414930

**Published:** 2025-01-22

**Authors:** Woynshet Yimer, Lakew Asmare, Fikre Bayu Gebeyehu, Tihtna Alemu, Anisa Mehamed, Fanos Yeshanew Ayele

**Affiliations:** ^1^Department of Public Health Nutrition, School of Public Health, College of Medicine and Health Sciences, Wollo University, Dessie, Ethiopia; ^2^Department of Epidemiology and Biostatistics, Institute of Public Health, College of Medicine and Health Sciences, University of Gondar, Gondar, Ethiopia; ^3^Department of Anatomy, School of Public Medicine, College of Health Sciences, Addis Ababa University, Addis Ababa, Ethiopia; ^4^Department of Surgical Nursing, School of Nursing, College of Medicine and Health Science, University of Gondar, Gondar, Ethiopia; ^5^Department of Epidemiology and Biostatistics, School of Public Health, College of Medicine and Health Sciences, Wollo University, Wollo, Ethiopia

**Keywords:** waist circumference, central obesity, factors, non-communicable disease, Eastern Ethiopia

## Abstract

**Introduction:**

A widely recognized public health issue affecting people worldwide is central obesity. Numerous chronic conditions, such as type 2 diabetes mellitus, cardiovascular disease, and malignancies are linked to this syndrome. There is limited information about waist circumference (WC) and its related variables among urban bank workers in Ethiopia. Therefore, the purpose of this study was to evaluate waist circumference and the factors associated with it among bank workers in Northeast Ethiopia.

**Methods:**

An institution-based cross-sectional study was carried out in Dessie, Northeast Ethiopia, between 2 October 2023 and 24 October 2023. A random selection method was employed to select 363 bank workers. STATA version 17 was used for analysis after the data were imported into EpiData version 4.4.2.0. Univariate and multivariate linear regression analyzes were performed to identify factors related to waist circumference. Normality, homoscedasticity, significant outliers, and multicollinearity were assessed, and a *p*-value of less than 0.05, along with a 95% confidence interval, was considered statistically significant.

**Results:**

A total of 345 participants were included in the final analysis, with a 95% response rate. The overall mean ± standard deviation (SD) of the waist circumference of the employees was 81.7 ± 6.8 cm, with 82.4 ± 6.5 cm for men and 80.7 ± 7.2 cm for women. The overall prevalence of central obesity was 57.7%, with 48.2% for the men and 70.3% for the women. The overall mean ± SD of the waist-to-hip ratio (WHR) was 0.90 ± 0.05, with 0.91 ± 0.04 for men and 0.89 ± 0.05 for women. The average waist circumference was significantly associated with the participants’ age (0.2 cm per year (SE: 0.1)) and MET hours (0.2 cm (SE: 0.1)). The mean waist circumference was 2.7 cm (SE:0.8) higher in the married women, 4.6 cm (SE:1.9) higher in the participants with non-communicable diseases (NCDs), and 1.7 cm (SE:0.8) higher in the participants who consumed discretionary calories for 4 days/week.

**Conclusion:**

The mean waist circumference among bank employees was higher, with more than half of the participants having central obesity. Age, marital status, discretionary calorie intake, non-communicable diseases, and metabolic equivalence task hours were the significant factors of waist circumference. Therefore, promoting activities aimed at preventing non-communicable diseases, such as leisure-time physical activity, and reducing discretionary calorie intake are essential for reducing high waist circumference measurements.

## 1 Introduction

Obesity, defined by waist circumference (WC) and measured midway between the lowest rib cage at the mid-clavicular line and the anterior superior iliac spine, is the most practical indicator of fat distribution and central obesity (1). It is the most common condition that causes disability, disease, and premature death and is measured using body mass index (2). The National Cholesterol Education Program Third Adult Treatment Panel (NCEP-ATP III) defines central obesity as a waist circumference of ≥102 cm for men and ≥88 cm for women, while the International Diabetes Federation (IDF) establishes cut-off points of ≥94 cm for men and ≥80 cm for women (3, 4). Additionally, specific cut-off points for Ethiopians are 83.7 cm for men and 78 cm for women (5).

A complex combination of behavioral, environmental, social, and hereditary factors contribute to obesity (3, 6). Being overweight or obese has been estimated to cause 35.8 million (2.3%) disability-adjusted life years and 2.8 million deaths worldwide each year (7). Sub-Saharan Africa has been facing the double burden of communicable and non-communicable diseases in recent years (8). Compared to BMI, central obesity is more strongly correlated with diabetes mellitus (DM) and cerebrovascular disease (CVD) (9). According to a linear dose–response analysis, the incidence of CVD increased by 3.4% for women and 4.0% for men with every 10 cm rise in waist circumference (WC) (10). The World Health Organization lists obesity as the fifth and seventeenth major risk factor contributing to the overall burden of disease in emerging nations with low and high death rates, respectively (11). Obesity has been linked to approximately 44% of diabetes cases, 23% of ischemic heart disease cases, and 7–41% of certain cancer cases (12).

Chronic health issues such as type 2 diabetes and cardiovascular diseases have been on the rise in many developing and underdeveloped nations, including Ethiopia, as a result of obesity and overweight. In Ethiopia, central obesity and overweight have become serious public health concerns, particularly among urban residents (13, 14). Ethiopia is currently facing the fallout from epidemiologic, demographic, economic, and nutritional shifts that continue to drive the spread of chronic non-communicable disease (NCD) epidemics (15). The burden of chronic disease morbidity and mortality is still rising despite government efforts to reduce risk factors for chronic diseases, such as central obesity (16).

There have been very few studies conducted on the prevalence of obesity based on body mass index in our country (17). Work environments that require limited physical activity (18), such as banks, are major employers. Thus, the purpose of this study was to evaluate obesity among urban bank employees in the study area. The results of this study can serve as a guide, especially for individuals who are at high risk.

## 2 Methods

### 2.1 Study design and setting

An institution-based cross-sectional study was conducted from 2 October to 24 October 2023 among urban bank employees in Dessie. Dessie is a city located 400 km to the north of the capital, Addis Ababa. It has a population of more than 230,733 people across five sub-cities (19). The city has 23 banks, with a total of 1,870 permanent employees, of whom 1,174 are men and the remaining 696 are women.

### 2.2 Study participants

The study population consisted of individuals aged ≥18 years, randomly selected from among the bank employees during the data collection period.

### 2.3 Inclusion and exclusion criteria

All permanent bank employees were included in the study, whereas pregnant women, mothers with <6 months postpartum, individuals with deformities on their backs, individuals unable to stand in an erect position, those with chronic edema, and non-permanent employees (employees with less than 6 months of work experience and outsourced security workers) were excluded from the study.

### 2.4 Sample size and sampling procedure

The sample size was calculated using the mean waist circumference formula, as the single population proportion formula and the double population proportion formula had lower sample sizes compared to using the mean. Therefore, with a standard deviation (SD) of 13.9 cm, a maximum accepted margin of error of 1.5 cm [taken from a previous study (20)], and a 95% CI, the final sample size, allowing for a 10% non-response rate, was 363.

**Figure 1:**
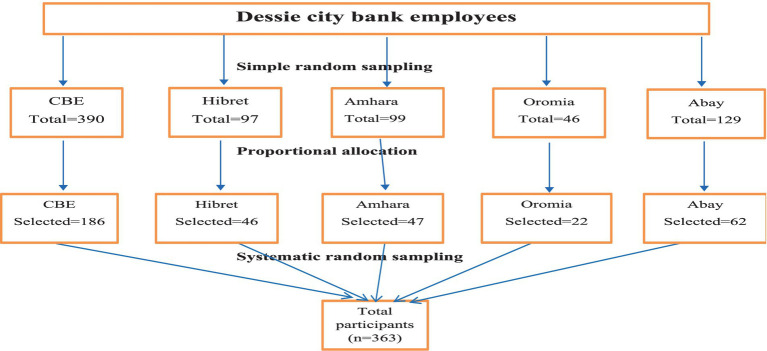
Schematic representation of the sampling procedure for waist circumference and the associated factors among the bank employees in Northeast Ethiopia, 2023.

There are 23 banks in Dessie city. Of these, five banks (Hibret, CBE, Amhara, Oromia, and Abay banks) were randomly selected. Then, an exhaustive list of permanent employees from the selected banks was prepared, and the number of the included participants from each bank was allocated proportionally. Finally, the participants were selected through systematic random sampling with a *k*-value of 2 (N/n = 761/363 ≈ 2). The first participant was selected using simple random sampling (lottery method), and the subsequent participants were selected at every second interval (Figure 1).

### 2.5 Measurement of variables

#### 2.5.1 Central obesity

For Ethiopian adults, it was defined as a waist circumference of 83.7 cm for men and 78 cm for women (21). Another cut-off point for Ethiopian adults was a waist-to-hip ratio (WHR) of 0.88 for men and 0.82 for women (5).

#### 2.5.2 Dietary diversity score

The dietary intake of the study participants was evaluated using a 24-h dietary recall. From the morning of the previous day to the morning of the current day, the participants were asked to list everything they had eaten, including snacks and food consumed outside their houses. The FAO’s nine food groups were used to classify these foods. Based on the number of food groups consumed, the participants were scored as being in the low, medium, or high range, with low being equal to or less than 3, medium being equal to or less than 4–5, and high being equal to or more than 6 (20).

#### 2.5.3 Servings of fruit and vegetables

After calculating the average daily consumption of fruits and vegetables, two groups were created, with the cut-off point being the mean consumption value of 0.95 servings/day (22).

#### 2.5.4 Alcohol consumption

The frequency of alcohol consumption was recorded, ranging from daily to less than once per month, based on the subjects’ consumption over the previous 12 months (23).

#### 2.5.5 Discretionary calorie intake

Discretionary foods include items such as soft drinks, cakes, Chocolate, and other high-sugar, low-nutrient food items that are also high in saturated fat, added sugars, and added salt (24).

### 2.6 Data collection procedure and tools

The data collection tool was designed according to the WHO STEP-wise approach to non-communicable disease surveillance and the Food and Agriculture Organization assessment guidelines (25). The questionnaires regarding demographic data, behavioral assessments, and physical assessments in the survey were completed. Four trained clinical nurses, two men and two women, collected the data under the direction of a healthcare professional holding a Master of Public Health degree. Using a constant tension tape, the waist circumference was measured to the nearest 0.1 cm at the midpoint between the top of the iliac crest and the lower edge of the last perceptible rib. Similarly, the hip circumference was measured at the level of the greater trochanter, or the widest part of the gluteal muscle, recorded to the nearest 0.1 cm.

### 2.7 Data quality assurance

Before the data were collected, the questionnaire was written in English, translated into Amharic, and then back into English using a different translator to maintain the questionnaire’s consistency. Training for supervisors and data collectors lasted 2 days. Before the actual data collection, 5% (19) of the final sample size had the questionnaire pretested on bank workers.

### 2.8 Data processing and analysis

EpiData version 4.4.2.0 was used to enter the obtained data, and the data were exported to STATA version 17 for analysis. Tables and figures were used to present the results of the computation of descriptive statistics. For each explanatory variable, univariate linear regression analysis was performed first, and multivariate linear regression analysis was then performed for variables with *p*-values less than 0.20. Multicollinearity in the multivariate linear regression model was assessed using the variance inflation factor (VIF) (values <10 for individual variables and <5 for the global value). The level of significance was set at a *p*-value of < 0.05.

## 3 Results

### 3.1 Sociodemographic characteristics

A total of 345 participants were included in the analysis, with a 95.0% response rate. The mean age of the study participants was 34.4 ± 6.7 years. The majority of respondents (216) were in the age category of 30–44 years. Only 27 (7.8%) participants had a tertiary education, and 42 (12.2%) held managerial positions (Table 1).

**Table 1:** Sociodemographic characteristics of the study participants among the bank employees in Northeast Ethiopia, 2023 (*n* = 345).

Variables	Categories	Frequency (%)
Sex	Men	197 (57.1)
	Women	148 (42.9)
Age	18–29	98 (28.4)
	30–39	216 (62.6)
	40–49	31 (9.0)
Current position	Officer	225 (65.2)
	Coordinator	78 (22.6)
	Manager	42 (12.2)
Marital status	Married	210 (60.87)
	Unmarried	135(39.13)
Level of education	Below degree	4 (1.2)
	Degree	314 (91.0)
	Above degree	27 (7.8)
Family size	1	110 (31.9)
	2–4	139 (40.3)
	≥5	96 (27.8)
Monthly income	<19,000	86 (24.9)
	19,000–25,999	78 (22.6)
	26,000–35,999	94 (27.3)
	≥36,000	87 (25.2)

Degree = Those who have completed grade 12.

### 3.2 Behavioral and personal health-related factors

Nearly all (99.4%) participants were non-smokers, and 204 (59.1%) had never consumed alcohol. The mean metabolic equivalent of task (MET)-min ± standard deviation (SD) was 203 ± 211, with only 18 (5.2%) participants meeting the target of 600 MET-min/week. Nearly all (99%) participants were non-smokers and 92.7%of the participants slept for 8 h/day. Only 13 (3.8%) of the participants had been diagnosed with non-communicable diseases (NCDs) (Table 2).

**Table 2:** Behavioral and personal health-related characteristics of the bank employees in Northeast Ethiopia, 2023 (*n* = 345).

Variable	Category	Frequency (%)
Ever smoked a cigarette	No	340 (98.55)
	Yes	5 (1.45)
Ever chewed chat	No	322 (93.3)
	Yes	23 (6.7)
Currently chewing chat (*n* = 23)	No	17 (73.91)
	Yes	6 (26.06)
Duration of chewing chat (*n* = 23)	<12 months	11 (47.8)
	≥12 months	12 (52.2)
Average grams of chat chewed (*n* = 23)	<100 gram	5 (21.7)
	≥100 gram	18 (78.3)
Ever consumed alcohol	No	141 (40.9)
	Yes	204 (59.1)
MET-min/week		203 ± 211
	Not meeting (<600 MET)	327 (94.8)
	Meeting (≥600 METs)	18 (5.2)
Time spent sitting (hours)		8.2 ± 1.2
	≤8	264 (76.5)
	>8	81 (23.5)
Time spent watching TV or using the computer (hours)		2.7 ± 0.7
	≤2	144 (41.7)
	>2	201 (58.3)
Time spent sleeping (hours)		8.5 ± 0.9
	≤7	25 (7.3)
	≥8	320 (92.7)
Having NCDs	No	332 (96.2)
	Yes	13 (3.8)
Type of NCD (*n* = 13)	HTN	8 (61.5)
	DM	4 (30.8)
	Kidney disease	1 (7.7)
Ever used contraception (*n* = 148)	No	51 (34.5)
	Yes	97 (65.5)
Length (years) of contraception use (*n* = 97)		2.9 ± 3.1
	≤2	53 (54.6)
	>2	44 (45.4)

Values are numbers (percentages) or means ± SD as appropriate; HTN, Hypertension; DM, Diabetes mellitus.

### 3.3 Nutritional factors

The mean ± SD of the servings of fruit and vegetables consumed per day was 0.95 ± 0.69, with 57.1% of the participants exceeding the mean value. The majority, 286 (91.0%), of participants had a medium DDS value, and 94 (27.3%) reported being currently fasting (Table 3).

**Table 3:** Nutrition-related characteristics of bank employees in Northeast Ethiopia, 2023 (*n* = 345).

Variable	Category	Frequency (%)
Servings of fruit and vegetables consumed/day		0.95 ± 0.69
	<0.95	197 (57.1)
	≥0.95	148 (42.9)
Protein source from animals/week or days	<4	293 (84.9)
	≥4	52 (15.1)
Protein source from plants /week or days	4	55 (15.9)
	≥4	290 (84.1)
Starchy staples/week or days	<4	49 (14.2)
	≥4	296 (85.8)
Milk and dairy products/week or days	<4	320 (92.8)
	≥4	25 (7.3)
Fats and oil products/week or days	<4	5 (1.5)
	≥4	340 (98.5)
Discretionary calories/ days	<4	245 (71.0)
	≥4	100 (29.0)
Fried foods/week or days	<4	338 (98.0)
	≥4	7 (2.0)
Types of oil commonly used	Liquid vegetable oil	266 (77.1)
	Others	79 (22.9)
Meals not prepared at home/week	<3 meals	268 (77.7)
	≥3 meals	77 (22.3)
Usually skips meal	Yes	93 (27.0)
	No	252 (73.0)
Currently fasting	Yes	94 (27.3)
	No	251 (72.7)
DDS	High	28 (8.1)
	Medium	286 (91.0)
	Low	31 (9.0)

Other*: Solidified vegetable oil, butter, margarine/peanut butter, shenolega, or none.

### 3.4 Waist-to-hip ratio and hip circumference

**Figure 2:**
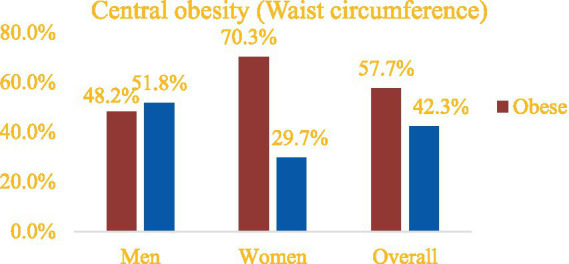
Prevalence of central obesity among bank employees in Northeast Ethiopia based on WC, 2023 (*n* = 345).

The overall mean ± SD of the waist circumference of the bank employees was 81.7 ± 6.8 cm. The mean WC was higher among men (82.4 ± 6.5 cm) than women (80.7 ± 7.2 cm). Accordingly, the overall mean ± SD of the WHR was 0.90 ± 0.05, with men having a value of 0.91 ± 0.04 and women having a value of 0.89 ± 0.05. The prevalence of central obesity was 57.7% (95% CI, 52.4–62.8%) (Figure 2).

**Figure 3:**
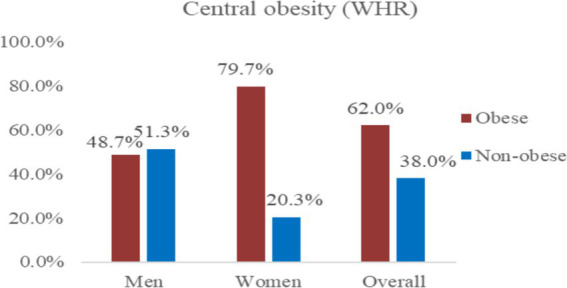
Prevalence of central obesity among bank employees in Northeast Ethiopia based on the WHR, 2023 (*n* = 345).

Similarly, the prevalence of central obesity based on the WHR was 62.0% (95% CI, 56.8–67.0%) (Figure 3).

### 3.5 Factors associated with waist circumference

In the univariate linear regression analysis, age, sex, position, marital status, family income, khat chewing, discretionary calorie intake, time spent sleeping, having NCDs, number of meals eaten outside the home/week, meal-skipping habits, and MET-hours were considered. The variables with *p*-values less than 0.2 were then included in the multivariate linear regression analysis (Table 4).

**Table 4:** Univariate and multivariate linear regression of factors associated with waist circumference among bank employees in Northeast Ethiopia, 2023 (*n* = 345).

		Waist circumference (cm)								
		Univariate linear regression					Multivariate linear regression			
		Slope					Slope			
	*n*	*r* ^2^	*b*	±	SE	*p*-value	*b*	±	SE	*p*-value
Total	345									
Sex		1.5%								
Men	197		1.7	±	0.7	0.02	1.3	±	0.7	0.08
Women	148		(80.7)				ref			
Age	345	9.5%	0.3	±	0.1	<0.001	0.2	±	0.1	0.01^*^
Position		4.9%								
Manager	42		3.5	±	1.1	0.002	1.1	±	1.4	0.43
Coordinator	78		3.0	±	0.9	0.001	1.2	±	1.0	0.26
Officer	225		(80.6)				ref			
Marital status		7.0%								
Married	210		3.7	±	0.7	<0.001	2.7	±	0.8	0.001^*^
Unmarried	135		(79.4)				ref			
Monthly family income		5.3%								
<19,000	86		(80.1)				ref			
19,000–25,999	78		0.1	±	1.0	0.96	−1.5	±	1.0	0.13
26,000–35,999	94		2.4	±	1.0	0.02	−1.6	±	1.2	0.18
≥360,000	87		3.7	±	1.0	<0.001	−2.6	±	1.5	0.09
Chat chewing		0.8%				0.11				
No	322		(81.5)				ref			
Yes	23		2.4	±	1.5		2.1	±	1.4	0.14
Discretionary calorie intake/week		2.1%								
≤3 days/week	245		(80.9)				ref			
≥4 days/week	100		2.5	±	0.8	0.002	1.7	±	0.8	0.03^*^
Time spent sleeping (hours)	345	0.6%	0.5	±	0.4	0.20	0.7	±	0.4	0.05
Having NCDs		2.7%								
No (%)	332		(81.5)				ref			
Yes (%)	13		5.9	±	1.9	0.002	4.6	±	1.9	0.01^*^
Number of meals/weeks	345	1%	0.5	±	0.3	0.07	0.4	±	0.3	0.19
Usually skips meal		1.2%								
Yes (%)	93		(80.4)				ref			
No (%)	252		1.7	±	0.8	0.04	0.8	±	0.8	0.31
Physical activity (MET-hours)	345		−0.2	±	0.1	0.02	−0.2	±	0.1	0.03^*^
Constant							66.5	±	3.7	

^*^p-value < 0.05; SE, Standard Error.

Thus, a one-year increase in age was associated with Intermediate increase of 0.2 cm (Standard error (SE): 0.1) in WC. In addition, the mean WC was 2.7 cm (SE:0.8) higher in married women compared to unmarried women. Similarly, the participants who consumed discretionary calories for 4 days/week had Intermediate WC increase of 1.7 cm (SE:0.8) compared to those who consumed discretionary calories for 3 days/week. Moreover, participants with NCDs had Intermediate WC value of 4.6 cm (SE:1.9) higher than their counterparts. A one-unit increase in MET-hours was associated with Intermediate decrease of 0.2 cm in the WC (SE:0.1).

Regarding the standardized beta coefficient, marital status and age had a greater effect on the waist circumference compared to the other significant variables. Accordingly, a change of one standard deviation in age resulted in a 0.18 standard deviation increase in the waist circumference. Similarly, on average, the married participants had a 0.19 standard deviation increase in the waist circumference compared to the unmarried participants.

## 4 Discussion

The mean ± SD values of the WHR and WC were found to be 0.90 ± 0.05 cm and 82.4 ± 6.5 cm, respectively, based on the results. The prevalence of central obesity was 57.7% based on WC and 62.0% based on the WHR. The average waist circumference was 81.7 ± 6.8 cm (95% CI: 81.0–82.4 cm). Compared to women [80.7 ± 7.2 cm (95%CI, 79.5–81.9)], men reported higher WC values [82.4 ± 6.5 cm (95%CI, 81.5–83.3 cm)]. In comparison to studies conducted in Australia, Costa Rica, and Indonesia, the mean waist circumference (WC) in this study was lower. In Australia, the mean WC was 97.5 cm for men and 87.5 cm for women (26); in Costa Rica, it was 86.4 cm for women and 88.1 cm for men (27); and in Indonesia, it was 92.4 ± 1.3 cm for men and 89.5 ± 3.2 cm for women (28). This discrepancy may be attributed to the higher intake of processed foods, sugar-sweetened beverages, and sedentary lifestyle in developed countries (29, 30). However, a lower mean WC was reported in Mozambique, with values of 75.2 cm for women and 76.1 cm for men (31). This disparity could be explained by differences in the study time, study environment (urban vs. both urban and rural), and study design (community-based vs. institution-based). Furthermore, the main causes for the various WC reports are national variations in sociocultural, economic, and behavioral norms. Conversely, similar results were found in a survey conducted in Addis Ababa, Ethiopia (81.8 cm for men and 80.7 for women) (20). This could be a result of the research’s comparable metropolitan settings and similar study periods. Based on the study’s findings, each additional year of age was linked to an average increase of 0.2 cm. Scientific data indicate that aging is linked to an increase in abdominal obesity, with a mean rise of 2.43–2.68 cm in visceral adipose tissue (32). In addition, metabolic rate, muscle mass, and bone mass decrease as age increases (33, 34). These findings are supported by related similar studies from South Africa (35), Korea (36), Iran (37), Benin (38), Southwest Ethiopia (39), Western Ethiopia (40), Eastern Ethiopia (41), and Adama town, Ethiopia (42), which reported a positive association between age and central obesity.

Married participants had a significantly higher average waist circumference value than the unmarried participants. This result is in line with studies conducted in China among twins (43), Greece (44), Western Ethiopia (31), Eastern Ethiopia (20), and Adama town, Ethiopia (45). This may be due to changes in behavior and lifestyle that occur after marriage, which affect energy consumption and expenditure. Moreover, married individuals may engage in less daily activities and exercises as parents often have less time for such activities (46, 47). The level of physical activity, computed as MET-hours, had a significant association with WC. This finding is supported by studies conducted in Ethiopia (14), Sri Lanka (48), Benin (49), Iran (50), Southwest Ethiopia (39), and Eastern Ethiopia (41). This may be because physical activity helps in reducing belly fat and increases total energy expenditure. The most changeable aspect of energy expenditure is leisure-time physical activity, and workplace physical activity may lower the risk of obesity in adults (51).

The study found a strong correlation between waist circumference and discretionary calorie intake each week. This result is consistent with other research findings, which reported that adults who consumed fewer discretionary calories had noticeably smaller waist circumference compared to adults who consumed more calories (52). This may be because discretionary foods are high in calories, low in nutrients, and high in saturated fat, added sugars, added salt, and cholesterol (48, 53). In addition, compared to their counterparts, individuals with NCDs had a noticeably higher average WC value (54, 55).

### 4.1 Strengths and limitations of the study

The study addresses an important gap by investigating central obesity in a specific occupational group (bank employees) in Ethiopia, a setting with limited prior research. The inclusion of diverse variables (e.g., dietary diversity, physical activity, and discretionary calorie intake) allowed for a robust multivariate analysis of central obesity determinants. The lack of detailed hip circumference analysis may have diminished the validity of the WHR findings. In addition, establishing causal inference was not possible due to the cross-sectional nature of the study. Furthermore, causal inferences could not be established due to the cross-sectional design of the study. Furthermore, height and weight data were not collected for the study group, allowing us to assess the limits of their influence on our findings.

## 5 Conclusion

Because bank personnel had a high mean waist circumference value, almost half of the participants were classified as centrally obese. Significant variables that influenced the waist circumference included age, marital status, discretionary calorie intake, the presence of NCDs, and MET-hours. These results highlight the necessity of implementing initiatives specifically designed to prevent non-communicable diseases by encouraging physical activity, promoting exercise during leisure time, building physical fitness facilities, and raising awareness of the importance of a healthy diet. All of these efforts can contribute to reducing the burden of obesity in the general population.

## Data Availability

The original contributions presented in the study are included in the article and further inquiries can be directed to the corresponding author.
